# From Wide Cognition to Mechanisms: A Silent Revolution

**DOI:** 10.3389/fpsyg.2018.02393

**Published:** 2018-12-06

**Authors:** Marcin Miłkowski, Robert Clowes, Zuzanna Rucińska, Aleksandra Przegalińska, Tadeusz Zawidzki, Joel Krueger, Adam Gies, Marek McGann, Łukasz Afeltowicz, Witold Wachowski, Fredrik Stjernberg, Victor Loughlin, Mateusz Hohol

**Affiliations:** ^1^Institute of Philosophy and Sociology, Polish Academy of Sciences, Warsaw, Poland; ^2^Faculty of Human and Social Sciences, New University of Lisbon, Lisbon, Portugal; ^3^Department of International Management, Kozminski University, Warsaw, Poland; ^4^Department of Philosophy, The George Washington University, Washington, DC, United States; ^5^Department of Sociology, Philosophy and Anthropology, University of Exeter, Exeter, United Kingdom; ^6^Department of Philosophy and Religion, Clemson University, Clemson, SC, United States; ^7^Department of Psychology, Mary Immaculate College, University of Limerick, Limerick, Ireland; ^8^Institute of Sociology, Nicolaus Copernicus University, Toruń, Poland; ^9^Institute of Philosophy, University of Warsaw, Warsaw, Poland; ^10^Faculty of Arts and Humanities, Linköping University, Linköping, Sweden; ^11^Department of Philosophy, University of Antwerp, Antwerp, Belgium; ^12^Copernicus Center for Interdisciplinary Studies, Jagiellonian University, Kraków, Poland

**Keywords:** embodied cognition, grounded cognition, extended mind, scaffolded mind, enactivism, distributed cognition, mechanistic explanation, wide mechanism

## Abstract

In this paper, we argue that several recent ‘wide’ perspectives on cognition (embodied, embedded, extended, enactive, and distributed) are only partially relevant to the study of cognition. While these wide accounts override traditional methodological individualism, the study of cognition has already progressed beyond these proposed perspectives toward building integrated explanations of the mechanisms involved, including not only internal submechanisms but also interactions with others, groups, cognitive artifacts, and their environment. Wide perspectives are essentially research heuristics for building mechanistic explanations. The claim is substantiated with reference to recent developments in the study of “mindreading” and debates on emotions. We argue that the current practice in cognitive (neuro)science has undergone, in effect, a silent mechanistic revolution, and has turned from initial binary oppositions and abstract proposals toward the integration of wide perspectives with the rest of the cognitive (neuro)sciences.

## Introduction

Traditionally, cognitive science has been methodologically individualist and has treated cognition as the capacity of individuals. Usually, it has framed intelligent behavior in terms of the processing of internal representations of individual minds and explained it in terms of functional analyses of mental capacities. The epitome of such methodology can be found in the important milestone both for cognitive science and cognitive neuroscience, David Marr’s *Vision* (Marr, 1982).

Recently, *embodied* cognition, *embedded* cognition, the *extended* mind, *enactivism*, as well as *distributed* cognition, have offered challenges to the traditional approach in different ways. Broadly speaking, what connects these positions is that they account for cognition in terms of embodied interactions supported and extended by actively built cognitive niches. This variety of approaches, which we dub “wide cognition,” offer a new picture of cognition. Cognition is no longer understood in an extremely modular fashion, for example as opposed to perception, action, or emotion, and is situated in social and cultural contexts.

Yet by the same token, the controversies over these wide perspectives have become outdated. Distributed cognitive processing is no longer usefully understood in terms of embodied cognition, extended cognition, enactivism, or distributed cognition *alone*, and our claim is that these views, in essence, offer mainly abstract heuristics that cannot do much explanatory work in isolation. In this paper, we propose that they are more useful in offering guidance for building multifaceted models of causal mechanisms responsible for cognition. Indeed, by going mechanistic, wide approaches can become non-trivial and integrated explanatory proposals, and we believe that this is what is happening right now.

In this paper, we first concisely characterize the contribution of several of the wide perspectives that were essential to the mechanistic turn in the study of cognition, which we shortly summarize. Next, by relying on case studies, we show that none of these perspectives alone offers the whole explanatory framework for cognition. Instead, they offer heuristic guidance for the study of mechanisms involved.

## Wide Accounts of Cognition

By *wide cognition* we mean an approach to cognition that cites *wide* factors, i.e., factors that go beyond intracranial processes. These include embodied, embedded, extended, enacted, and distributed cognition, which are related but distinct concepts. These research approaches are also inspired by earlier wide accounts of cognitive processes that include ecological psychology (Gibson, 1986), biosemiotics (Favareau, 2010), and sociocultural psychology (Leont’ev, 1974; Vygotsky, 1986).

In contradistinction to traditional frameworks of cognitive science, these approaches do not explain cognitive phenomena solely (or at all) in terms of the intra-neural manipulation of (language-like) internal representations but stress the fact that (a) minds can extend into or rely heavily on the environment; (b) embodiment is essential to cognition; (c) cognition is enacted, or constructed, in an active fashion; (d) cognitive phenomena are always constituted by interactions with the environment; (e) and cognitive acts are not always but sometimes paradigmatically distributed among multiple agents. However, theoretical presentations of these approaches remain fairly abstract and focused on deciding yes–no questions rather than building unified models of cognitive phenomena.

In other words, the accounts of wide approaches follow the steps of “trying to play 20 questions with nature and win,” as Newell (1973) has put it. Newell observed that cognitive psychologists had their tasks of choice that they treated as indicative of grand issues in psychology, but their theorizing remained bizarrely tied both to details of single tasks and to grand issues at the same time, without painting any broader picture of cognition. These grand issues were stated in an extremely abstract fashion: nature vs. nurture, continuous vs. all-or-none learning, serial vs. parallel processing, analog vs. digital, conscious vs. unconscious, grammars vs. associations for language, etc. Similarly, in the debates over wide cognitive approaches, instead of explaining how cognition works by describing underlying and interacting mechanisms, at least some researchers attempt to win the debate by showing that the bodily, the environmental, or the interactive aspect is most essential in cognitive functioning. This did not work in the 1970s (as Newell rightly noted) and remains unproductive today. Grand issues in the study of cognition cannot be fruitfully understood in terms of a series of simple dichotomies.

However, wide approaches also inspired researchers to search for more integrated explanations, just like abstract questions paired with cherry-picked experimental phenomena that earlier (negatively) inspired Newell to propose the study of unified architectures of cognition. Simply, Newell thought that an integrative theory was lacking in cognitive psychology (Newell, 1973, 1990). As we argue, mechanistic explanations are used to the same effect today: to offer an integrated view on cognition, but in a piecemeal fashion, unlike Newell who promoted the idea of sketching a general blueprint for all cognitive systems.

### Embodied and Grounded Cognition

The claim of *embodied cognition* (EC) is that the physical body of an agent is relevant to cognition; in other words, cognitive processing includes, in a non-trivial fashion, not only processes that occur in the brain but also processes that occur outside the brain, in the body of the agent.^1^ Cognition thus depends deeply on the features of the physical body (Varela et al., 1991; Clark, 1997; Damasio, 2000; Gallagher, 2005; Byrge et al., 2014; Shapiro, 2018).

EC has become increasingly widespread in all areas of cognitive science—from neuroscience and cognitive psychology to cognitive linguistics, philosophy, computer science, and robotics. Over the past decades, this has led to an impressive burst of experimental evidence for the role of the body in cognition, in particular within psychology and neuroscience (Willems and Hagoort, 2007; Barsalou, 2008; Fischer and Zwaan, 2008; Toni et al., 2008; Jirak et al., 2010; Pulvermüller, 2013; Costello and Bloesch, 2017; Varga and Heck, 2017). The shared aspect of EC theories is their opposition to classical, propositional views, which hold that cognition boils down to the manipulation of amodal, abstract, and arbitrary codes (cf. Shapiro, 2014). In contrast, according to EC theorists, cognition is constrained and enabled by the specific characteristics of our own brain-body system.

### Embedded and Situated Cognition

The *embedded and situated* approach to cognition holds that cognition should be framed in terms of the (usually time-pressured) interaction of the agent and its immediate surroundings. The extra-bodily context constrains and enables cognition (Agre and Chapman, 1987; Suchman, 1987; Norman, 1993).

Many lines of inquiry offer support to the embedded mind (and by extension, situated cognition). For example, behavior-based robotics, as pioneered by Brooks (1991), arguably supports the claims made by embedded cognition and situated cognition. Brooks demonstrated that it was possible to build robots capable of performing simple tasks despite those robots having no detailed, internal knowledge of the environments in which they were operating. Such robots were designed to be “set up to be set off” by certain features of their local surroundings, bypassing the need for complex, internal cognitive machinery. The work of Brooks, and many others, has shown that basic cognitive functions could be causally dependent on movement and structures in the local environment (Steels and Brooks, 1995).

### Extended Mind

The *extended mind* is the idea that an agent’s mind is not necessarily brain-bound and can incorporate external resources such as tools, language, and external systems to enhance or augment cognitive processes (Clark and Chalmers, 1998). This idea may be understood narrowly to mean that that some elements of the agent’s surrounding are its proper cognitive parts, while according to a broader account, a hybrid system is constituted by coupling the agent’s cognitive processing with its environment (Clark, 2008b; see also Hutchins, 2014).

The difference between this approach and embedded cognition lies in the claim, endorsed by the extended mind theorists but rejected by embedded cognition theorists, that at least some parts of what was traditionally considered as ‘the environment’ may be properly understood as literal parts of the agent’s mind.

The practical implications of the extended mind turn on exactly how and under what circumstances external resources might integrate with internal or biological ones. What properties of cognitive artifacts might make them prone to integration? Theorists differ in how they answer this question (Sterelny, 2010). Some have endorsed an extended functionalist approach (Clark, 2008b; Sprevak, 2009; Wheeler, 2017), whereby it is the functional (i.e., causal) role of the technology that makes it part of someone’s mind. Others insist that it is the bodily manipulation of such technologies that integrates them into a cognitive routine (Menary, 2010b). A related but importantly distinct approach is the complementarity framework (Sutton, 2006; Menary, 2010a), which proposes that we tend to integrate tools and artifacts into our cognitive processes when they provide resources which complement our existing biological systems. Menary’s idea of cognitive integration comes, in fact, very close to the idea of multiscale and multilevel causal explanations as required by mechanistic explanation.

### Enactive Cognition

The *enactive* approach to cognition recognizes a crucial inter-dependency between an autonomous agent and the world it inhabits. Cognitive activity is wholly determined neither by the agents nor by their environment, but rather it emerges from the inter-dependency between the two.

This observation lies at the heart of the enactive approach. It forms the basis for the mode of analysis and understanding central to enactive thinking (Thompson, 2007; McGann et al., 2013). However, one can distinguish at least four quite distinct flavors of the enactivist framework: *sensorimotor enactivism*, which deems action as essential to perception and cognition (O’Regan and Noë, 2001); *autopoietic* (or classical) enactivism, which grounds cognition in autonomous organization of biological entities (Varela, 1979, 1997; Weber and Varela, 2002); *participatory sense-making* enactivism, which understands cognition as relying on interactions between autonomous agents (De Jaegher and Di Paolo, 2007; Thompson and Stapleton, 2009; Di Paolo and De Jaegher, 2012); and *radical enactivism*, which denies the role of mental representation in explanations of cognition (Hutto and Myin, 2013). Just because the sensorimotor flavor is conceptually close to embodied cognition, it may be considered as an action-oriented version of embodied cognition. The empirical consequences of radical enactivism are still a matter of some dispute. Thus, for the sake of simplicity, below, we focus on the classical autopoietic version of enactivism and its later extension, participatory sense-making enactivism.

For enactivists who build on the work of Varela et al. (1991), cognition is a process of *sense-making*, a continuous process of adaptive coping by an agent to the vagaries of being a living being embedded in a complex world (Thompson and Stapleton, 2009). In this sense, cognition is directly continuous with the biological processes of staying alive in what is sometimes called a “life-mind continuity” (Thompson, 2007). An organism makes sense of its world through the process of integrating the environment effectively into its own processes of self-production. In the domain of chemistry and biological cells, this process of self-production is called “autopoiesis” (Maturana and Varela, 1980; Weber and Varela, 2002; Froese and Stewart, 2010), and this capacity for continued self-production provides a ground-level value to the activity of the system. But the same dynamics, if different processes, apply at other levels of analysis, such as the production of identities within social groups. These different levels of description are never wholly independent of one another, because all cognitive activity is bodily, and so values that arise within the social domain are always contextualized and coupled (even if loosely) to values within the biological (Di Paolo et al., 2017).

Social interaction is a special case of such engagement with the environment, in which the agent’s activity is not simply coordinated with a physical world but with another agent. Sense-making in such situations is not simply coordinated but negotiated, a process termed *participatory sense-making* (De Jaegher and Di Paolo, 2007). Where effective social coordination is achieved, participatory sense-making produces meaning and cognitive activity that is shared across the participants. This sense-making must be understood as much in terms of the conversation or interaction as a whole as through the combined activities of the individuals involved.

### Distributed Cognition

The *distributed cognition* (DC) approach expands the classical focus on cognition as a property of an individual organism/agent toward the components and operations of larger cognitive systems, which may encompass multiple individual agents and artifacts.

This distinguishes DC from other approaches labeled here as ‘wide cognition,’ which usually treat an individual organism as the main unit of analysis, describing, for example, the way an individual is embedded, connected to his/her environment or how his/her body matters from the perspective of cognitive processes. In the case of DC, the focus is on heterogeneous elements (e.g., biological, material, discursive), which take part in sequential or simultaneous processes of generation, transmission and transformation of representational states. DC identifies such processes with cognition (Hutchins, 1995a). Moreover, in DC, representational states are not understood as mental states or other inner states of any individual agent. Examples of representational states can instead include meaningful gestures or poses of agents, written or spoken information, visualizations displayed on screens, lines drawn on a navigational chart and the chart itself, but also non-symbolic cues which modify the behavior of agents (Zhang and Norman, 1994). Representatives of DC stress that a larger cognitive system works in a different manner and has different properties than individual agents who may be part of the system. Unlike the extended mind approach, the agent’s mind need not be the center of such a cognitive system: “some systems have a clear center while other systems have multiple centers or no center at all” (Hutchins, 2014, p. 37). However, according to the pioneer and leading theoretician of the approach, Edwin Hutchins, DC is not a kind or a special case of cognition, but a perspective on all of cognition; therefore, “all instances of cognition can be seen as emerging from distributed processes” (Hutchins, 2014, p. 36), which is especially useful in studying a particular type of systems mentioned above. Thus, the difference between the extended mind approach and DC is not a difference of type, but a difference of the level of the analysis.

## From Wide Perspectives to Mechanisms

Even if one can analytically distinguish embodied, embedded, extended, enacted, and distributed views, they are not always mutually exclusive,^2^ and a large body of research exhibits properties specific to many of the above approaches. This is not a coincidence. They are not poised to be complete and exclusive accounts of cognition. They are not theories in the sense of providing complete predictions or explanations of phenomena in question. For this, they are too abstract. There is no particular novel prediction that the embodied perspective may offer when applied, for example, to group decision making in a faculty meeting. In this, these perspectives are not different from grand research traditions of cognitive science.

### Providing Guiding Heuristics

Wide approaches are research traditions, and research traditions are not to be conflated with complete theories. To illustrate this, take computationalism, which is another grand research tradition in cognitive science (Miłkowski, 2018). As such, however, computationalism does not offer any predictions for group decision making either. What they do instead is provide certain guiding heuristics. A proponent of traditional computational modeling would ask what the overall task is and why solving it is appropriate; what the algorithms and representations involved are; and how they are physically implemented. The embodied cognition paradigm asks how this task is related to bodily, in particular to sensorimotor, features of decision makers. The embedded approach points out that there may be important environmental factors, and the extended mind may alert us to the fact that there might be important cognitive artifacts in operation. Finally, the enactive perspective (at least in its non-classical version) points to participatory negotiation of how the activity is perceived by various agents involved, and the distributed perspective hints that the phenomenon may involve not only human agents but also external representations and instruments. Note that even if the phenomenon is only slightly influenced by bodily features, for example, it need not mean that embodied cognition is thereby falsified. Heuristics are fallible, after all.

We claim that instead of asking yes/no questions to nature, at least some researchers involved in the study of cognition from wide perspectives are more and more interested in building models of cognitive mechanisms. This kind of explanatory practice goes beyond mere binary oppositions that state that the role of the environment is crucial or not, or that the environment has been appropriated by the cognitive system as its proper part. Instead, these researchers treat—or at least should treat—cognitive systems as organized spatiotemporal systems, comprised of entities and activities that are jointly responsible for their phenomena of interest, which is evidenced by attempts to integrate, for example, embodied and extended approaches (Clark, 2008b; Borghi and Cimatti, 2010; Borghi et al., 2013; Walter, 2014). Differences between approaches matter only for expository purposes but not really for their practice, which involves mechanistic modeling of cognition.

### Relying on Mechanisms

As proponents of so-called *new mechanism* stress, many fields of science already appeal to mechanisms to explain their phenomena of interest (Machamer et al., 2000; Glennan, 2017). This is true of life sciences but also cognitive (neuro)science (Thagard and Kroon, 2006; Waskan, 2006; Bechtel, 2008; Miłkowski, 2013; Piccinini, 2015) and social sciences (Hedström and Ylikoski, 2010). While the philosophical analyses of the notion of mechanism differ (Glennan and Illari, 2017; for a review, see Illari and Williamson, 2011), in a nutshell, the idea is the following: A mechanism is an organized spatiotemporal structure responsible for the occurrence of at least one phenomenon to be explained. The orchestrated causal interaction of the mechanism’s component parts and operations explains the phenomenon at hand. In a certain sense, the new mechanistic account is extremely lean, leaving out practically all physical details of what mechanisms as such might be. They are only causally organized spatiotemporal structures.

According to the mechanistic account of explanation, there are no mechanisms *per se*, only mechanisms *of* some phenomena; mechanisms should not be confused with organized spatiotemporal systems, processes or structures. Mechanisms are wholes comprised of component parts and operations but they are delineated by the phenomena they are responsible for, and *phenomena* are not to be conflated with observable features of a given spatiotemporal system; to understand the nature of a phenomenon, extensive theoretical considerations may be required (Bogen and Woodward, 1988).^3^ For example, it is widely agreed that human beings are capable of producing a potentially unbounded number of linguistic utterances (Chomsky, 1959). Obviously, it is physically impossible to observe anyone producing literally an infinite series of utterances because people are mortal. But there are theoretical reasons to specify the phenomenon of linguistic productivity in such an idealizing fashion.

### Constitutive Explanations

Mechanistic explanations that elucidate how a given phenomenon occurs by referring to component parts and operations of a mechanism are called *constitutive explanations.* Explanatory texts of this kind cite the internal causal and part-whole organization of the mechanism as responsible for the phenomenon at hand. For example, to explain the phenomenon of how one cuts a piece of rope with scissors, we can describe the scissors as composed of two metal blades with handles connected so that the sharpened edges slide against each other when handles are closed. In other words, there are components (blades with handles, a screw that joints the blades) and operations (bringing together the blades) organized in such a way that cutting occurs.

Given the importance of the study of components and operations, it is not surprising that mechanistic explanations are guided by two important heuristics: localization and decomposition (Bechtel and Richardson, 2010). By localizing where operations happen and breaking down the whole system into its component parts, researchers discover the internal structure of the mechanism. Importantly, a larger mechanism may comprise a number of individual mechanisms organized together; while the circulatory system is a mechanism responsible for blood circulation, its component mechanism, the heart, is also a mechanism, which is a proper part of the circulatory system. The goal of mechanistic modeling is to be able to conceptually recompose the mechanism from its component parts and operations. Recomposing is only possible when the explanatory text is complete, that is, when it satisfies the completeness norm. This norm requires that the explanatory text represent “all and only the relevant portions of the causal structure of the world” (Craver, 2007b, p. 27). This is not to say that mechanistic explanations are supposed to give every possible detail about the mechanism; no, only explanatorily relevant detail counts (Baetu, 2015; Craver and Kaplan, 2018).

Localization and decomposition are merely heuristics; they may fail without making mechanistic explanation impossible. Bechtel and Richardson stress that while “decomposability may be a natural and fruitful starting point, it may be no more than that” (Bechtel and Richardson, 2010, p. 32). Fully decomposable systems are an extreme case, in which the sum is nothing more than its parts. As Bechtel and Richardson stress, it is much more likely that biological systems are near decomposable (Bechtel and Richardson, 2010, p. 26). The notion of near decomposability was introduced by Herbert A. Simon, who stressed that the behavior of near decomposable component systems in the short run is approximately independent from the behaviors of other systems, and in the long run, it depends only in an aggregate way on the behavior of the other components (Simon, 1962).^4^ Some biological systems may be more highly integrated, which does not make them resistant to mechanistic explanations, as some critics claim (Anderson, 2015). This is because mechanisms may include a highly complex organization of their internal component parts and operations. Such mechanisms, however, may be much more difficult to study and could require the use of specific mathematical techniques developed for research on complex systems.

In particular, the dynamic approach to cognition may stress systemic interactions in cognitive systems, but they need not exclude the possibility of providing dynamical and mechanical explanations. As many have argued (Kaplan and Bechtel, 2011; Zednik, 2011; Kaplan, 2015; Lyre, 2017), some phenomena require the use of mathematical methods typical of dynamical systems to build complete mechanistic causal explanations.

### Role of the Environment for Mechanisms

Part-whole relationships and the causal structure of the mechanism are usually not sufficient to wholly explain the phenomenon at hand: the explanation must include crucial environmental modulation. Only in some very special circumstances, where the whole dynamics of the phenomenon depends merely on the underlying mechanism, can one ignore the environmental modulation (as Craver (2007a) does). In the case of scissors, one has to include fingers that are required to move the blades if one is to give a complete explanation of cutting. However, fingers are not parts of scissors because they are not constitutively relevant to cutting. As Carl Craver has argued, a good criterion of what counts as a component *x* of a mechanism *S* is its constitutive relevance in the operation of *S* (for example, cutting a piece of rope is an operation of scissors). A component *x* of *S* is constitutively relevant for *S* if and only if there is a relationship of mutual manipulability between *x* and *S*:

(i) *x* is part of *S*; (ii) in the conditions relevant to the request for explanation there is some change to *X’s φ*-ing that changes *S*’s *ψ*-ing; and (iii) in the conditions relevant to the request for explanation there is some change to *S*’s *ψ*-ing that changes *X*’s *φ*-ing. (Craver, 2007b, p. 153).

Even if we consider fingers to be spatiotemporal parts of whatever cuts the rope, they normally do not fulfill condition (iii): even if moving fingers does change how scissors’ blades move, blades themselves do not change fingers (or at least, not much; the application of the physical force will of course impact fingers as well). Note: it is, in principle, possible to build mechanisms that would work this way (some kind of self-organizing scissors with robotic fingers attached, for example); it just doesn’t work this way in our simple case, in which we count fingers as environmental modulators. In other words, mechanisms are not conceived as exclusively responsible for their phenomena.^5^ On the contrary, mechanistic explanations routinely include the environment and interactions with other mechanisms.

Mechanistic explanatory strategies have been nicely summarized by Bechtel in his metaphor of looking down, around and up:

Accounts of mechanistic explanation have emphasized the importance of looking down—decomposing a mechanism into its parts and operations. (…) But once multiple components of a mechanism have been identified, researchers also need to figure out how it is organized—they must look around and determine how to recompose the mechanism. (…) Researchers also need to look up—situate a mechanism in its context, which may be a larger mechanism that modulates its behavior. When looking down is combined with looking around and up, mechanistic research results in an integrated, multi-level perspective (Bechtel, 2009, p. 543).

What we stress in this paper is that mechanistic approaches do not focus narrowly on the internal structure of cognitive agents; these approaches require “going wide” to include causally relevant parts of the environment and interactions with other cognitive agents. In fact, the accounts of explanation defended by new mechanists are already wide. Although it may seem that, to explain something mechanistically is to elucidate only its internal mechanism, it is not correct in cases where recomposition of the phenomenon requires additional explanatory factors. Quite naturally, this happens in most biological and cognitive cases. For this reason, wide mechanistic explanations can be used by all researchers interested in the interaction of cognitive systems with their environments, as embedded cognition would have it. Mechanistic explanations can be also applied to study the cases of extended cognition and analyze the boundaries of cognitive systems (Kaplan, 2012), as we will show in Section “Applying a Mechanistic Approach to Mindshaping.”

### Bottoming Out: The Basic Level of Mechanistic Explanations

To add more explanatory depth to our toy example, one could inquire why the blades are made of metal rather than, say, paper or polypropylene. In this case, the physical structure of blades has to be included in the explanation. The constitutive explanation *bottoms out* at the level that is considered sufficiently well understood to elucidate how a whole mechanism operates. In a certain sense, therefore, mechanistic constitutive explanations are reductionist (Hensel, 2013) because they appeal to the internal causal structure of mechanisms to explain the phenomena at hand. However, they are not reductionist as they do not screen off complex interactions inside the mechanism and environmental modulations. In other words, the whole mechanism is not replaced in a constitutive explanation by an appeal to general laws of constituents of the mechanism. Moreover, the level at which an explanation bottoms out is decided largely pragmatically by the research community. Thus, the new mechanistic approach is reductionist only to a moderate extent and in a very extended sense, in which any denial of the absolute autonomy of psychology from neuroscience or from social science counts as reductionism. It is at the same time non-reductionist because it usually situates a given mechanism in a larger context (Wright, 2007).

In cognitive science, mechanistic explanations will therefore routinely involve interaction with the environment, cognitive performance as well as the neural underpinnings of this performance. Current cognitive science, in contrast to the evidential standards of the 1960s or 1970s, requires bottoming out at a level studied with neuroscientific methods (Boone and Piccinini, 2016); right now, it is not required to cite molecular or cellular data but evidence about neural structures responsible for psychological function is normally expected to make a given explanation plausible. The norms that govern where explanations bottom out are not, however, legislated *a priori* by philosophers in their armchairs, but are assumed as valid in research practices in various fields of inquiry, which may (sometimes implicitly) set their own differing standards. Note also that the detail added to bottom out the explanation has to be still explanatorily relevant; otherwise, it would violate the completeness norm. So, any detail that does not bring any further value has to be discarded.

The mechanistic approach is also natural in the study of relations between the physical body of an agent and its neural systems, which is the focus of embodied cognition. Although proponents of enactivism usually appeal to dynamical explanations (but see also Abramova and Slors, 2018), these explanations can be typically recast in mechanistic terms (Zednik, 2011). In addition, distributed cognition can be easily framed in terms of mechanistic explanations; the cognitive systems composed of cognitive agents and artifacts can be naturally understood as mechanisms comprising component parts and operations on external representations. In the next subsection, we describe distributed cognition as mechanistic more fully.

### Airplane Cockpit as Exemplar Cognitive Mechanism

The mechanistic approach can be illustrated with an example of a study about how the structure of the cockpit in an airplane supports complex, distributed cognitive processes. Edwin Hutchins studied in particular the use of physical devices to remember the speed of the aircraft:

The cockpit system remembers its speeds, and the memory process emerges from the activity of the pilots. The memory of the cockpit, however, is not made primarily of pilot memory. A complete theory of individual human memory would not be sufficient to understand that which we wish to understand because so much of the memory function takes place outside the individual. In some sense, what the theory of individual human memory explains is not how this system works, but why this system must contain so many components that are functionally implicated in cockpit memory, yet are external to the pilots themselves (Hutchins, 1995b, p. 286).

**FIGURE 1:**
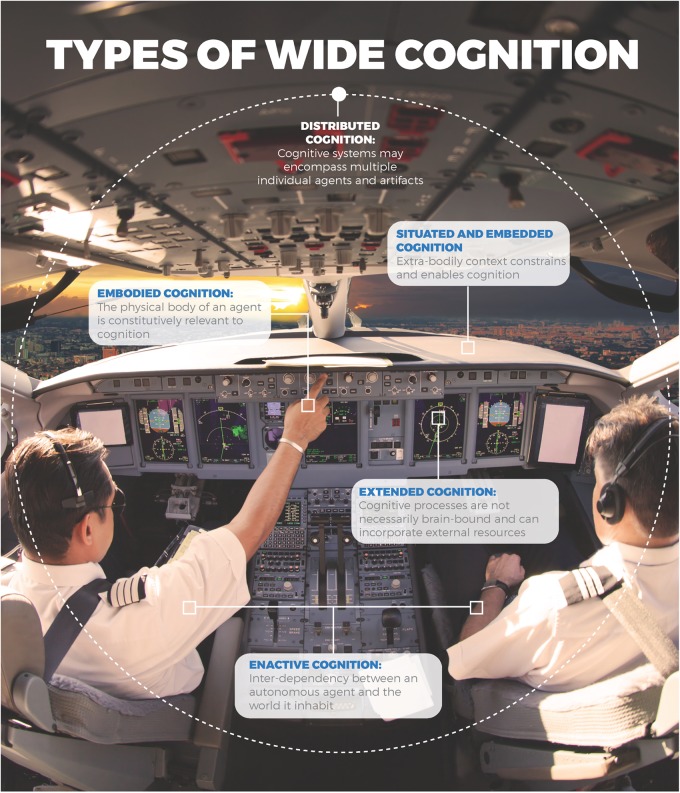
Wide approaches to the study of cognition.

The process is distributed and includes two pilots as its component submechanisms, one responsible for flying and navigating the plane, and another responsible for communication and other tasks. But the distributed approach may well appeal to heuristics preferred by other wide approaches to the study of cognition (see Figure 1).

This can lead to the discovery of all sorts of crucial mechanisms. First, the embodied perspective will hypothesize that the design of control devices is adapted to sensorimotor characteristics of a human being, and may study the quality of design in terms of how easily it can be operated, for example, by seeing whether surfaces become slippery, buttons difficult to press and confusing, etc. The situated perspective will insist that the cognitive artifacts in the cockpit all constrain, and help to determine the plane weight, etc., which is crucial to land the aircraft. Alternatively, the extended view may study how well the minds of individuals mesh with multiple devices on board. Lastly, the enactive perspective will stress the importance of dynamic coordination between pilots and consider how the environment is structured in terms of various affordances. At the same time, the distributed approach does not screen off the study of representational devices; on the contrary, as Hutchins stresses:

This article presents a theoretical framework that takes a socio-technical system, rather than an individual mind, as its primary unit of analysis. This theory is explicitly cognitive in the sense that it is concerned with how information is represented and how representations are transformed and propagated through the system. Such a theory can provide a bridge between the information processing properties of individuals and the information processing properties of a larger system, such as an airplane cockpit (Hutchins, 1995b, pp. 286–287).

This example shows how wide cognition, with its different theoretical component approaches that can cluster together, is poised to study phenomena related to cognitive processing. This suggests that specific wide perspectives are not particularly attractive in isolation. Instead, they can be further developed and integrated to form a larger mechanistic framework (cf. Abramova and Slors, 2018; Wachowski, 2018).

### Silent Mechanistic Revolution

The mechanistic revolution in cognitive science is silent insofar as it need not involve any widespread theoretical controversies. Contra to an opinion made popular by Kuhn (1962), a significant portion of *the* Scientific Revolution happened without much controversy (Wootton, 2015). Mechanistic explanation, as the underlying methodological framework, is not universally accepted in psychology, or at least in philosophy of psychology (Shapiro, 2016; Weiskopf, 2016). Most of the time, however, it goes unnoticed, just like references to mechanisms in papers and textbooks in cognitive science (for example, see how frequently Gardner (1985) mentions biological, cognitive, psychological, and information-processing mechanisms).

To further show that wide cognition is studied already in a mechanistic fashion, even if the notion of mechanism itself is not used prominently, we will describe two cases in some depth: we will look at explanatory accounts of mind-reading and emotion that go beyond a single ‘wide’ account of cognitive processes. We will show that in both of these cases, they can be understood as wide mechanistic explanations. They capture the essence of wide cognition.

## Wide Mechanisms of Mindshaping

In this section, we describe mind-reading as a multi-faceted phenomenon. One of the ways to frame mind-reading is to understand it in terms of mindshaping (Zawidzki, 2013), which has both institutional and biological underpinnings. The proposed explanation is best understood, we claim, in mechanistic terms, and cannot be accounted for as exemplifying a merely an embodied, embedded, extended, enactive, or even distributed perspective on cognition.

### Previous Models: Mindreading

In philosophy, as well as in developmental and comparative psychology, social cognition has traditionally been understood in terms of *mind-reading*. This is a technical term that is often (although not always) used interchangeably with “mentalizing” and “theory of mind.”

The guiding concept behind the notions of mind-reading, mentalizing, and theory of mind is that an individual’s success at predicting the behavior of and coordinating with her con-specifics depends on understanding the unseen psychological causes responsible for their behavior. There are two classic competing accounts of mind-reading: theory theory (Perner, 1991; Gopnik and Meltzoff, 1997) and simulation theory (Harris, 2000; Goldman, 2006). According to theory theory, mind-reading relies on a body of implicit knowledge, represented as general rules or underwritten by innate modular mechanisms. In contrast, simulation theory holds that understanding others does not depend on inferential processes relying on rules or modular mechanisms but on mental modeling, pretending or imagining oneself to be in the other’s situation. In spite of this difference, the two competing accounts share one important feature: on these orthodox conceptions of human social cognition, differences in the sophistication of social interaction, e.g., cooperation and coordination, across phylogenetic and ontogenetic time, can be traced to the sophistication of interactants’ theories of mind. For example, whereas non-human primates are restricted to an understanding of con-specifics’ goals, perceptions, emotional states, and behavioral dispositions, adult humans can also attribute beliefs, intentions, and other propositional attitudes, arranged in rational plans causally responsible for their interactants’ behaviors.

However, this orthodox understanding of social cognition remains under considerable pressure. For example, Dennett (1971, 1987) argues that a non-human animal, a human infant, and even a typical human adult operates with a form of social cognition that does not depend on their understanding of the unseen psychological causes of each other’s behavior via sophisticated theories of mind. Rather, he argues, most social cognition involves an understanding of perspectives—an ability to track relations between whole organisms and features of the environment salient to these organisms—what Dennett calls an “intentional stance” (cf. Zawidzki, 2012). Understanding of hidden psychological causes, especially propositional attitudes, depends on prior enculturation in culturally specific practices (Køster, 2017), and is useful only in making sense of deviations from or exceptions to typical behavioral patterns.

It is widely recognized that propositional attitudes, like belief and desire, bear very tenuous relations to observable circumstances and behavior. The reason is holism: even on orthodox views, particular propositional attitudes lead to behavior only against a background of indefinitely many other propositional attitudes (Bermúdez, 1995). For example, a belief that it is raining will issue in umbrella retrieval only when conjoined with a desire to stay dry that is stronger than competing desires, appropriate beliefs about the location of an umbrella and the relative costs of retrieving it, etc. This feature of propositional attitudes raises significant problems for the orthodox conception of social cognition as being dependent on mind-reading, and especially for the widely held assumption that successful navigation of the social world requires attributing propositional attitudes. This is because it raises issues of computational tractability. Most successful social interactions take place seamlessly and dynamically at relatively short time scales. It seems unlikely that successful interpreters search the immense space of propositional attitude attributions compatible with brief, observed bouts of behavior, in time to arrive at attributions accurate enough to support successful interaction (for a more detailed computational analysis, see Zeppi and Blokpoel, 2017). For this reason, a number of theorists have recently raised worries about the orthodox conception.

### Applying a Mechanistic Approach to Mindshaping

Zawidzki (2013) argues that human populations are distinguished from our closest non-human relatives by an array of effective “mindshaping” techniques, aimed at making potential interactants more alike, and hence easier to interpret, which is a clear example of “looking around” internal cognitive mechanisms, or adopting the situated perspective in the study of cognition. For example, only humans engage in “over-imitation” (Nielsen and Tomaselli, 2010), that is, copying each other’s fine-grained, apparently non-functional behaviors. Only humans engage in constant and pervasive pedagogy. Only humans set up elaborate normative regimes, and linguistic, narrative constructs, and then pressure each other to conform to them. Such practices tend to make human populations easier to interpret.

In addition, Zawidzki argues that the attribution of full-blown propositional attitudes evolved to play a justificatory, rather than a predictive, function. That is, rather than help predict the behavior of potential interactants, attribution of full-blown propositional attitudes evolved as a tool for situating interactants in a normative space—as committed and entitled to various discursive and non-discursive moves, given prior such moves. Inspired by Brandom’s (1994) theory of discursive practice, this idea finds some support in social psychology as well. Bruner (1990) points out that many everyday uses of propositional attitude attributions are triggered by behavior that deviates from a canonical cultural pattern, and function to excuse or at least make sense of the behavior, e.g., when a person appears to break some norm, as when one fails to fulfill a promise, sanctions can be mitigated if one can provide an excuse in terms of what one was thinking. There is some empirical evidence in favor of this hypothesis: Malle et al. (2007) showed that, when explaining behavior, adults are much more likely to appeal to reason explanations, i.e., propositional attitudes, when they are motivated to make the behavior look good. Without such motivation, they are more likely to explain behavior in terms of causal history.

### Incorporating Culture Into a Wide Mechanism

The mind-shaping hypothesis, and the role of propositional attitude attribution in justifying apparently counter-normative behavior to one’s interactants, suggest a very precise sense in which human social cognition is “wide”: much of the work required to predict our fellows involves not intracranial socio-cognitive resources, like so-called “theory of mind,” but rather, external cultural practices aimed at making our likely interactants more familiar and easier to anticipate. Culture seems to function as a classic example of “epistemic action” (Kirsh, 1996; Clark, 1997). Rather than tackle the seemingly computationally intractable task of predicting our con-specifics by building ever more complex, intracranial computational capacity, natural selection seems to have developed systematic means of structuring the social environment in ways that make it easier to predict using relatively simple intracranial resources. The variety of mind-shaping techniques employed by a given culture—over-imitation, pedagogy, norm construction and enforcement, the use of linguistic narrative, e.g., myths, to construct “virtual” models for all to imitate—can all be seen as forms of wide social cognition, aimed at regimenting the potentially unruly and intractable social environment. If this hypothesis is on the right track, then it suggests that the study of culture and its effects on cognition must play a central role in the sciences of social cognition.

What this hypothesis suggests, therefore, is that a wide array of factors—factors that go beyond properties of single individuals—have to be included in the explanation. But one cannot simply produce this explanation by referring merely to embodiment, embeddedness, cognitive extension or enaction, or even distributed cognition. These perspectives offer limited guidance for the case at hand. Instead, the focus on cultural and social factors is much more salient.

In fact, over the past 15 years there have been many new research programs exploring the impact of culture on the development of human cognitive structures. Areas of knowledge that deal with this subject are cross-cultural psychology, neuroanthropology and cultural neuroscience. What unites the above-mentioned disciplines is the emphasis put on socio-cultural aspects of human cognitive abilities. This position is sometimes known as bio-cultural constructivism (Baltes et al., 2006) and it states “that brain and culture are in a continuous, interdependent, co-productive transaction and reciprocal determination” (Baltes et al., 2006). The basic assumption is that the structure of the human brain is not programmed *a priori*, but rather is co-shaped by the environmental stimulation in a broad sense: the socio-cultural experience of the entity, its environment, etc. Generalizing, the process of ontogenetic development is stimulated genetically, environmentally and culturally. Consequently, this leads to the abandonment of radical genetic, neuronal, cultural determinism, or environmentalism and highlights the simultaneous impact of all the above factors on human ontogeny and evolution. This, in turn, means that researchers produce explanatory texts that mention complex mechanisms, whose causal organization may lead to observed cognitive performance.

### Relevance of the Mechanistic Approach

At the same time, the mindshaping hypothesis is right now not fully developed in terms of a complete mechanistic explanation. In particular, the entities and operations responsible for the cultural and social constraints on human behavior are not fully understood in terms of neurocognitive mechanisms. For this reason, this explanation remains highly schematic and so, according to the mechanistic view, requires further development. But this development clearly requires integrative efforts, which is what researchers studying mindreading usually presuppose. For example, it is most likely that separate mechanisms underlie the various forms of mindshaping, i.e., overimitation, pedagogy, norm institution and enforcement, and self-constitution in terms of roles in narratives expressed in public language. These mechanisms may be, in turn, involved either in attributing unobservable entities such as beliefs, or in mere tracking relational properties of bouts of behavior. The mindshaping hypothesis suggests the latter may be the case for a number of them (Fenici and Zawidzki, 2017). Decomposing the dense web of interrelationships between these mechanisms is also a difficult ongoing research task.

In other words, just like most mechanistic explanations in life and cognitive sciences, this explanation is far from the ideal; nevertheless, mechanistic norms of completeness may drive further study of the phenomena in question. From our point of view, it is crucial to stress that a wide perspective on mind-reading naturally fosters the development of mechanistic explanations.

## Emotions and Their Expressions

In this section, we show how the mechanistic view offers methodological advice and goes beyond idle debates over the role of the environment in emotional expression.

### Previous Models of Emotion

Dominant theories of emotion tend to adopt an individualistic perspective. Whether modeled as evaluative judgments, appraisal processes, physiological states of bodily arousal, or something else, emotions are commonly thought of as private states individuated by their neurobiology, cognitive content, behavioral expression, or phenomenal character (Damasio, 2000; Nussbaum, 2001; Prinz, 2004; Laird, 2007; Russell, 2009; Panksepp, 2014). From this perspective, the social and cultural environment is of secondary interest for understanding the inner (i.e., agent-centric) mechanisms that are the real heart of emotions. However, the mechanistic approach to explanation does not conform to this kind of individualism, even if bodily aspects of emotion are usually understood as individual.

### Role of Embodiment for Emotion

One can argue that the embodied character of emotions is a necessary condition on their being social. The embodied account of emotion claims that the brain alone is not sufficient to generate emotional experience. Rather, the rest of the (non-neural) body, in all its biological, physiological, morphological, and kinematic details, makes a non-trivial contribution to the realization of some emotions. For example, many studies appear to indicate a reciprocal relation between an emotional experience and its behavioral expression. Subjects induced to adopt an emotion-specific facial expression or posture report experiencing the corresponding emotion (Edelman, 1984; Duclos and Laird, 2001; Laird, 2007; Winkielman, 2010; Marzoli et al., 2013; Davis et al., 2015). Conversely, inhibiting the expression (e.g., suppressing the facial signature of anger or happiness) diminishes the associated experience (Strack et al., 1988; de Gelder, 2006; Niedenthal, 2007; Oberman et al., 2007; Niedenthal et al., 2009; see however Wagenmakers et al., 2016 for the results of replication of Strack et al., 1988). Individuals who suffer severe spinal cord injuries and lose the capacity to behaviorally express emotions report less-intense feelings of high-arousal emotions like fear and anger (Chwalisz et al., 1988; Laird, 2007; see Bermond et al., 1991). Still other studies have found that inhibited facial expressiveness—e.g., following Botox injections (Davis et al., 2010; Havas et al., 2010) or due to congenital facial paralysis (Cole and Spalding, 2008) — results in diminished emotional phenomenology.

Embodied approaches to emotion thus emphasize the extent to which emotions depend upon extra-neural factors and feedback (Winkielman et al., 2015). Moreover, if the physical expression of anger, say, is literally a part of the anger itself—that is, part of its physical realization—some emotions, in virtue of their embodied character, can be said to have a social face. They are partially constituted by world-directed features perceptually available to other agents (Krueger, 2012). But this embodied perspective remains a fairly conservative way of thinking about the social character of emotions because the environment does not enter into this characterization in any substantive manner.

### Incorporating Sociality Into Wide Mechanism of Emotion

A wide mechanistic perspective on emotions, by contrast, urges that emotions are fundamentally social phenomena: they are scaffolded and shaped by features of the agent’s social niche, as well as the various ways the individual—along with other emotional agents—actively modifies and negotiates this shared niche (Fischer and Manstead, 2005; Krueger, 2013; Slaby, 2014; Colombetti and Roberts, 2015; Krueger and Szanto, 2016; Colombetti, 2017). Accordingly, the role of the environment is seen not merely as providing stimulus inputs and serving as an arena for behavioral outputs. Rather, the environment—understood broadly to include not only material features of the agent’s niche, but also sociocultural and interpersonal aspects as well—plays an active role in shaping emotions on multiple time scales (Parkinson et al., 2005), including both the moment-to-moment character of emotional episodes, as well as the long-term development of an individual’s emotional repertoire (Griffiths and Scarantino, 2008). While not entirely jettisoning a consideration of internal mechanisms, a wide perspective on emotions thus argues that the larger bodily, social, and interactive context in which emotions are situated needs to be part of the target *explanandum*. This also follows naturally from the mechanistic perspective, which provides no privileged explanatory position for the biological agent. For mechanists, the set of all and only causally relevant factors relevant to a given phenomenon counts as explanatorily relevant.

To a large extent, proponents of embodied or extended accounts of emotion are not that radical in claiming that emotions cannot be understood exclusively as properties of individuals. From the evolutionary point of view, the expression of emotion plays primarily a communicative role (Darwin, 1872), and to understand the causal structure of the communication process, one cannot limit the explanation to the agent that experiences one emotion. Moreover, because they play communicative roles across various species, there is a strong selection pressure to develop at least a limited number of universal expressions of emotions, a point widely appreciated nowadays (Ekman, 2016; but see Barrett L.F., 2016 for a different view). For example, it has been elaborated in experiments that look at multiple sources of feedback that may modulate the felt emotion itself.

Consider the work on audience effects, which indicates that emotional responses differ, depending on whether there is an audience or not. For example, ten-pin bowlers smile significantly more after producing a positive event (e.g., bowling a strike or a spare) when they turn to face their friends than when they are still facing the pin (Kraut and Johnston, 1979). A similar effect has been observed in Spanish soccer fans who issue authentic (i.e., “Duchenne”) smiles (Ekman et al., 1990) in response to goals only when facing another person (Fernández-Dols et al., 1997), as well as in Olympic gold-medal winners who smile almost exclusively when receiving their gold medal (and not when they are alone prior to the ceremony) (Fernández-Dols and Ruiz-Belda, 1995). Audience effects have even been observed in young infants (Jones et al., 1991). Other work in developmental psychology demonstrates how the repertoire of physical strategies caregivers use to engage with infants—facial expressions, postural adjustments, exaggerated gestures and vocalizations, gaze manipulation, etc.—function as real-time scaffolding supporting the emergence, regulation, and performance of many basic emotions from the beginning of life (Tronick et al., 1978; Posner and Rothbart, 1998; Rochat, 2009; Reddy, 2010; Hobson, 2011). This research supports the thesis that certain emotions depend crucially upon the ongoing (i.e., synchronic) feedback and support of the social-communicative context in which they are situated.

### Relevance of the Mechanistic Approach

A mechanistic explanation of emotional phenomena need not deny the importance of the brain or individual mechanisms, and most emotions do not need to be actually perceived by some other agent to be felt. But it should avoid incomplete explanations, which by being excessively narrow in selecting causally relevant factors and abstracting emotions away from the broader bodily, social, and cultural contexts, violates an important mechanistic explanatory norm, namely completeness (Craver, 2007b). The modulating effects of emotional expression and feedback, however, cannot support the radical view that emotion itself extends into the physical environment. One way to justify this point is to appeal to methodological principles of what counts the part of a given mechanism (see Role of the Environment for Mechanisms above), which state that only what is constitutively relevant to the phenomenon to be explained is part of the mechanism.

Consider now the case of an Olympic medal winner. The environment, for example, seats on the Olympic stadium, would be constitutively relevant to the emotion as long as their removal would change the emotion, and if the change of the emotion of winners would influence the seats. This is definitely not the case; only the emotions felt by agents who perceive emotional expressions change. Moreover, a perception of the expressed emotion may trigger this (or another) emotion. Nonetheless, the mechanistic approach, while not without its own problems,^6^ can help to clarify the issues in the debate over the “wide” nature of emotions. The only candidate components for “extended emotional mechanisms,” beyond one’s own bodily and neural processes, are the bodily and neural processes of other agents, not arbitrary physical things in the environment.

Further, the explanatory advantage of assuming the extended mind or extended emotion, over the embedded account thereof, is rather doubtful.^7^ While it is certainly possible to adopt a view on emotional or cognitive phenomena that would make certain non-neural and non-bodily processes components of emotional or cognitive mechanisms, as long as the completeness norm is followed, this move makes no explanatory difference vis-à-vis considering the same phenomena more narrowly construed, this time just modulated by environmental triggers.^8^

Mechanistic criteria of constitutive relevance decide what is a component of a mechanism but not what a given mechanism is a mechanism of. This is up to a theorist. It’s a large oversimplification to say that the mechanism is question in the first case is just an “emotional mechanism”; it’s rather a mechanism of reciprocal influence of emotional expression, which may include a number of agents. In such a case, not only do both the embedded and extended view appeal to the same causal networks, while insisting that the boundaries of what they consider to be emotional mechanisms are different. But it would be mildly confusing to call these mechanisms simply “emotional” because their function can be specified more precisely and is context-bound. The debate based on oversimplification is thus idle; from the mechanistic point of view, both parties are wrong because the phenomena to be explained are not just emotions. Nevertheless, the mechanistic approach to explanation does not decide where the mind or emotion starts or ends.

To sum up, in this section, we have argued that the wide perspective on emotions requires more conceptual clarity, and this is what the mechanistic approach offers. It can resolve some conundrums by dissolving explanatorily idle debates. While further research on emotions as relying on non-individual factors is required by mechanistic norms of explanation, in particular to fully link social factors with mechanisms studied by affective neuroscience, the mechanistic approach aptly describes current integrative efforts in the study of emotion.

## Possible Objections

In this section, we briefly review possible objections to the claim that there is an ongoing mechanistic revolution in wide approaches in cognitive (neuro)science.

### Dynamical, Not Mechanistic

One way to argue against the claim that mechanisms underlie the ongoing scientific practice in cognitive (neuro)science, in particular inspired by wide perspectives, is to say that dynamical explanation is more frequently appealed to by its proponents. Indeed, Menary (2007), in proposing his framework of cognitive integration, which takes inspiration from the extended and situated perspectives, stresses the importance of dynamical explanation:

it is based on the idea of multiple cognitive layers where neural, bodily, and environmental processes all conspire to complete cognitive tasks. Although the framework is unified by a dynamical systems description of the evolution of processing in the hybrid and multi-layered system, it recognizes the novel contributions of the distinct processing profiles of the brain, body, and environment (Menary, 2015, p. 2).

There are two possible lines of reply to this argument. The first one is to stress that *mere* dynamical explanations are actually explanatorily unsatisfactory (Kaplan and Craver, 2011). In fact, dynamical explanation at its core relies on the received view of explanation, which requires appeal to universal laws (Hempel and Oppenheim, 1948, 1953). When a dynamical explanation is not equivalent to a mechanistic one, it is, according to Craver and Kaplan, simply deficient because it is open to well-known objections put forward against the received view (cf. Craver, 2007b). Dynamical regularities referred to in cognitive explanations are usually not universal generalizations and remain invariant in an extremely limited number of contexts. Although one could in principle re-describe a mechanism by appealing to regularities, these regularities will be true of entities and activities whose organization is jointly responsible for a given phenomenon. It is extremely difficult to find dynamical explanations that do not appeal to causally organized systems; and this is what makes purported dynamical explanations equivalent to mechanistic explanations (Walmsley, 2008; Zednik, 2011). In other words, as far as explanations involved in Menary’s cognitive integration framework are genuine, they are, despite appearances, mechanistic.

Another but related reply is to stress the role of activities or processes, which makes dynamical explanations part and parcel of a particular kind of explanations, namely dynamical mechanistic explanations (Bechtel, 2015). In other words, the mechanistic approach to explanation sometimes has to appeal to dynamical models, in particular when time-related phenomena are in question.

To wit, the proponent of dynamicism has the burden of proving that mechanistic explanations are somehow deficient. But this is extremely difficult. All successful dynamical explanations can be exploited for building mechanistic explanatory models, while rejecting dynamical attempts that violate mechanistic norms (for example, they abstract away from the rich internal structure of mechanisms when it is causally relevant). By way of reply, the dynamicist could claim that these purported non-mechanical dynamical explanations are crucial in cognitive science by systematically reviewing the most significant empirical results achieved in the last 50 years and showing that they cannot be properly understood in either functionalist or mechanistic manner. Still, until the dynamicist does this, both of the aforementioned responses are available to the mechanist.

### Functionalist Alternative

Opponents of mechanistic explanation may further object that mechanisms are not the sole focus of cognitive science; in particular, they may claim that functional explanations are genuinely explanatory (Shapiro, 2016). However, one may counter that mere functional analyses are no longer accepted in cognitive science as satisfactory and are in fact treated as mere mechanism sketches, or essentially incomplete explanations (Piccinini and Craver, 2011). Nonetheless, mechanistic explanations in cognitive science are in one respect close to the functional approach in that they deal with mechanisms that have biological or psychological functions (Garson, 2013), but in fact wide perspectives are incompatible with the autonomy of psychology presupposed by functional explanation traditionally understood. Functional autonomy was sometimes spelled out in extreme terms and this extreme appeal to autonomy is what the mechanistic approach rejects. For example, autonomy was defended by saying that we could be made of Swiss cheese, and it wouldn’t matter (Putnam, 1975, p. 291). It would; we wouldn’t be human cognitive agents anymore, just inert blobs of cheese. The causal organization of an agent cannot be duplicated in just any physical substrate. Similarly, scientists no longer accept abstract box-and-arrows diagrams as satisfactory explanations.

One major difference between functionalist box-and-arrows diagrams and mechanistic explanations is that most defenders of functionalism required only that the posited functional organization be sufficient for the capacity to be present (Newell and Simon, 1972, p. 13; Cummins, 1975). For example, according to defenders of sufficiency analysis, one could produce a computer simulation of translation that would behave similar as human translators, and such simulation would be explanatory. However, critics point out that the same behavior could be produced in different ways and that genuinely explanatory models have to be constrained by what is known to be not only sufficient but also actually causally relevant (Miłkowski, 2013, p. 119). In the case of machine translation, statistical and neural-network methods might produce similar outputs but the first one is not even remotely biologically plausible (Koehn, 2010). This is why abstract diagrams of systems sufficient to perform some activity are no longer considered explanatory in contemporary cognitive research.

To prove this point, one can point out that the currently influential predictive processing account of cognition, which remains largely sketchy and devoid of detailed models of the causal dynamics of the nervous system, strives for mechanistic evidence (Gordon et al., 2017). Were neuroscientific evidence not useful, the effort in producing it would be a symptom of irrationality among researchers, and defenders would not take pride in showing this kind of evidence. Contrarily, it is exactly the fact that the evidence about entities and activities is required to substantiate functional analyses that makes it also a mechanistic sketch. These are current evidential standards in cognitive (neuro)science (Boone and Piccinini, 2016).

Second, as soon as one adopts an embodied and situated perspective, autonomy claims and functionalism cease to be attractive. As proponents of embodiment should be aware, autonomy claims were not successfully established by recourse to multiple realization, or the (purported) fact that some capacities may be realized by any system with the same functional organization but sufficiently different causal structure (cf. Aizawa and Gillett, 2011; Polger and Shapiro, 2016). It is simply much more natural to adopt a mechanistic perspective and to argue for embodiment than to adopt functionalism and argue for embodiment, even if many proponents of embodied cognition adopted a functionalist perspective (Clark, 2008a). If bodily features could be multiply realized by just any functional structure, are they actually *bodily* features? For example, it seems highly dubious that a computational simulation of a body might replace the physical bodily interaction in any biological agent without loss, and the stress on the role of the physical body is at the core of a strong embodiment thesis (Dempsey and Shani, 2013).

A related objection may be connected to the fairly abstract and sketchy nature of some proposed wide explanations. For example, the notion of affordance, which has become popular among wide approaches, is usually introduced in terms of agent-environment interaction, without positing internal mechanisms at all. But cognitive neuroscientists do not consider it absurd to inquire into neurocognitive mechanisms of affordance perception; in fact, there is already some work consistent with a mechanistic approach (Young, 2006; Cisek, 2007).

In other words, it seems that the overarching assumption in cognitive science today is that one cannot simply point to mere functional analysis. Mere functional analysis is essentially incomplete because it need not contain any relevant causal detail: sufficiency analysis may produce structures that have no causal relevance for the phenomenon, as in the case of traditional statistical machine translation, which is wildly disparate from how people translate even if its results may fairly coincide.

### Anti-representationalism and Anti-computationalism

In some discussions about wide approaches to cognition, it is presupposed that they exclude representationalism or computationalism (Barrett L., 2016), which have been traditional assumptions of mainstream cognitive science. But things are perhaps not so simple. Computationalism is certainly compatible with wide approaches, in particular when paired with the mechanistic account of physical computation (Miłkowski, 2013; Villalobos and Dewhurst, 2017), but the issue of representation is more complex. While proponents of radical enactivism remain highly critical of the notion of mental content (Hutto and Myin, 2013), their criticism, from the methodological point of view, is mostly motivated by parsimony arguments. In fact, in some cases it may indeed be worthwhile to see whether full-blown mental content is required for explanations instead of reliance on affordances in the environment, without deciding *a priori* that unobservable entities are not admissible in science, which admittedly would be an extreme interpretation of radical enactivism (Clowes and Mendonça, 2016; Gärtner and Clowes, 2017). Other proponents of wide approaches, particularly in the case of the perceptual symbol systems, remain strongly motivated by representationalism (Barsalou, 1999); many stress that wide approaches cannot and should not reject representationalism (Schlosser, 2017; Wheeler, 2017).

If wide approaches are understood as only offering generic heuristic advice, as we claim, then such approaches are not decisive when it comes to determining the status of computations or representations. Nonetheless, wide approaches can lead us to be careful when positing such processes, which is exactly what mechanistic explanation requires, namely that we need additional causal evidence from lower levels of mechanistic organization in order to talk of computation and representation.

## Summary

Wide approaches to cognition cannot be applied to the study of cognition in isolation. Methodological and sometimes ideological controversies around embodiment and situatedness, for example, look outdated when we focus on current explanatory practice in cognitive science. Researchers appeal to wide factors merely as discovery heuristics. In essence, the mechanistic turn that is beginning to pervade wide approaches in cognitive (neuro)science is the natural next step of the mechanistic revolution already prevalent in cognitive (neuro)science (Boone and Piccinini, 2016).

Wide perspectives on cognition, we claim, are fruitful when applied together in the practice of building mechanistic models, which can be further constrained, for example, by available psychopathological, neurophysiological, psychophysiological, or psychological evidence. Taken in isolation, they offer very little theoretical advice.

A wide mechanistic perspective should not deny the significance of the brain or individual mechanisms, or it will prematurely advise against testing hypotheses about potentially relevant causal factors. If cognition is not only the result of individual innate cognitive processes but also of culturally-afforded competence acquired by individuals and groups alike, it is only natural to assume that mechanistic explanations will include a fair number of wide causal factors. Wide cognition is therefore not a grand theory of everything that could supply all possible detailed hypotheses about cognitive phenomena. Instead, it merely helps reject self-imposed and unnecessary restrictions in the study of their mechanisms. We claim that the silent mechanistic revolution ongoing in cognitive (neuro)science helps to bring the insights from these wide perspectives together by showing their role as research heuristics.

## Author Contributions

MMi, RC, ZR, AP, TZ, JK, AG, MMc, ŁA, WW, FS, VL, and MH reviewed the literature, developed the theoretical stance, wrote the manuscript, and reviewed and accepted its final version.

## Conflict of Interest Statement

The authors declare that the research was conducted in the absence of any commercial or financial relationships that could be construed as a potential conflict of interest.

## Footnotes

1 Some authors (Barsalou, 2008, 2016; Pezzulo et al., 2011) reduce the stress put on the role of the body, and prefer to use the more general term “grounded cognition” in order to emphasize the fact that cognition is grounded, not only in bodily states, but also in situations, situated simulations, and so forth.

2 The embedded view may be considered inconsistent with the extended mind as far as these views do not agree what is the part of a cognitive system and what is not; a smartphone is not part of a cognitive system for a theorist of situated cognition, while it is literally part of one’s mind according to the extended mind thesis. But these frameworks are explanatorily equivalent from the mechanistic point of view, as we argue below. Here, we focus on their explanatory virtues.

3 Note that while theorists are free to specify phenomena according to their interest, they may also be wrong (Craver, 2009). For example, in the 19th century, American psychiatrists would diagnose “drapetomania,” or the desire to break free, as a psychiatric condition in slaves, while this phenomenon is not a psychiatric condition at all. It was simply a racist construct and causal models of drapetomania would fail to distinguish the purportedly pathological behavior from rational action because fleeing is rational for anyone kept in slavery (Pöyhönen, 2013).

4 Some analyze the notion of *modularity* in terms of near-decomposable systems (cf. Callebaut et al., 2005). Note however, that such notion is much more liberal than Fodor’s (1983) notion of modularity. Fodor’s account is geared towards cognitive phenomena and relies on such notions as domain specificity, which have no direct counterpart in mechanistic decomposability. Moreover, large-scale brain networks, partially overlapping and partially dissociable, may exhibit near decomposability to some extent, without being fully modular in Fodor’s sense. Thus, mechanistic explanation does not imply modularity in Fodor’s sense.

5 Moreover, a similar phenomenon may be produced in a different way, for example, using a knife to cut the rope. In complex biological cases, there may be even several compensatory mechanisms that evolved to produce similar phenomena in the case of damage of a basic mechanism. However, because a mechanism is specified by its phenomena from the mechanistic point of view, different ways of achieving even a very similar end would count as different mechanisms. We thank one of the reviewers for requiring more clarity about this point.

6 The issue of delineating boundaries of mechanisms in all possible cases goes far beyond the scope of this paper.

7 For further discussion of the explanatory equivalence of both accounts, see (Barker, 2010; Sprevak, 2010). It is argued that there is no matter of fact that could adjudicate between the two hypotheses.

8 If the phenomenon to be explained is considered “the reciprocal influence of agents’ emotional expression,” then one must include all and only mechanisms relevant for this process, which will include at least two agents. However, if one is interested only in the other agent’s felt emotions, then the first agent can be treated as the environmental trigger. The causal networks in both cases are different and depend (partially) on one’s explanatory interest. This is not to say that causation is not objective. What depends on our cognitive interests is how we specify the phenomena to be explained. Note, however, that some phenomena can be specified falsely, and some specifications can lead to overly complex or intractable models. There is no mechanism of telekinesis, for example.
